# Platycodon D reduces obesity and non-alcoholic fatty liver disease induced by a high-fat diet through inhibiting intestinal fat absorption

**DOI:** 10.3389/fphar.2024.1412453

**Published:** 2024-07-17

**Authors:** Xingkui Tang, Yi Yang, Wenxu Peng, Mengping Xu, Qitong Fan, Feng Li, Guorong Zou, Jianlin Zhu

**Affiliations:** ^1^ The Affiliated Panyu Central Hospital, Guangzhou Medical University, Guangzhou, China; ^2^ Cancer Institute of Panyu, The Affiliated Panyu Central Hospital of Guangzhou Medical University, Guangzhou, China; ^3^ Department of Bariatric Surgery, The First Affiliated Hospital of Jinan University, Guangzhou, China; ^4^ Guangzhou Medical University, Guangzhou, China; ^5^ Department of General Surgery, First Affiliated Medical Department, Gannan Medical University, Ganzhou, China; ^6^ Department of Anatomy, Fuzhou Medical College, Nanchang University, Fuzhou, China; ^7^ Infectious Diseases Institute, Guangzhou Eighth People’s Hospital, Guangzhou Medical University, Guangzhou, Guangdong, China; ^8^ Department of Endoscopy, Guizhou Provincial People’s Hospital, Guiyang, China

**Keywords:** intestine, lipid absorption, non-alcoholic fatty liver disease, platycodin D, obesity

## Abstract

**Background:**

Platycodin D (PD) has been reported to treat metabolic diseases, including non-alcoholic fatty liver disease. In addition, platycodin D has been reported to activate intestinal 5'AMP-activated protein kinase (AMPK) phosphorylation levels, thereby reducing lipid absorption. Therefore, the aim of this study is to explore whether PD activation of intestinal AMPK and reduced lipid absorption can improve non-alcoholic fatty liver disease.

**Methods:**

Clean-grade male C57/BL mice were fed a high-fat diet (HFD) (containing 60% calories) for 16 weeks, and oral PD (10 mg/kg/day) was administered at the same time. The liver and intestines were the collected, and the intestines were tested. The expressions of lipid absorption genes (CD36, NPC1L1, and ApoB), the serum total triglyceride (TG) and total cholesterol (TC) levels in the intestines and livers, the fecal free fatty acid (FFA) levels, and the expression of AMPK phosphorylated proteins in the intestines were examined using Western blot analyses. The lipid distribution in the livers, intestines, and fat was detected using Oil Red O and hematoxylin and eosin (H&E) staining. A colon cancer cell line (Caco2) was used to confirm the effect of PD on the cellular lipid uptake *in vitro*. In addition, serum inflammatory factors and liver enzymes were measured to clarify the impact of PD on the circulation of metabolic syndrome. Leptin-deficient mice (OB) were then used to further explore the improvement of PD on body weight and blood lipids.

**Results:**

PD had a very significant therapeutic or preventive effect on metabolic syndrome and fatty liver induced by a high-fat diet. PD improved body weight, insulin sensitivity, and glucose tolerance in mice fed a high-fat diet and also prevented non-alcoholic fatty liver disease, reduced blood lipid levels, and increased fecal lipid excretion. In addition, PD reduced lipid absorption by activating the intestinal AMPK protein, which may have involved the inhibition of the gene expression levels of intestinal lipid absorption genes (CD36, NPC1L1, and ApoB). The combined effect of these factors improved hepatic lipid accumulation and lipid accumulation in adipose tissue. It was further found that PD also improved the body weights and blood lipid levels of leptin-deficient mice (OB) mice.

**Conclusion:**

PD had a very strong therapeutic effect on mice under a high-fat diet. PD reduced high-fat diet-induced obesity and non-alcoholic fatty liver disease by inhibiting intestinal fat absorption.

## 1 Introduction

Platycodon is a perennial herb of the Campanulaceae family, and its root (Platycodi radix) is used as a medicinal herb in Northeast Asia and has therapeutic effects on respiratory diseases such as bronchitis, tonsillitis, sore throat, asthma, and tuberculosis (Lee et al., 2012; Zhang et al., 2015; Su et al., 2022). Modern research has shown that PG primarily contains triterpene saponins, flavonoids, phenolic acids, and other substances. Among these, platycodon D (PD) is an oleanane-type triterpenoid saponin and one of the primary active substances of PD (Jeon et al., 2017; Xie et al., 2023).

Many studies have shown that PD can activate the AMPK protein, thereby stimulating systemic metabolic switches and improving the body’s metabolic capacity (Shen et al., 2023). In addition, there are some reports that PD can improve the intestinal inflammatory response by activating AMPK (Wang et al., 2022). It has been reported that the activation of the AMPK protein in the intestine will cause the weakening of lipid absorption, which has the effect of improving body metabolism and reducing weight (Li et al., 2016; Kim et al., 2019). Therefore, we believe that the activation of the intestinal AMPK protein by PD may improve metabolic syndrome, including non-alcoholic fatty liver disease, insulin resistance, and white fat accumulation.

The annual incidence of non-alcoholic fatty liver disease (NAFLD) is increasing on a global scale, and it is a disease that endangers public safety (Wang et al., 2023; Xu et al., 2024a). In the past few years, many researchers have paid extensive attention to the treatment and prevention of NAFLD. Although NAFLD is increasingly common, treatment guidelines have not yet been developed. To develop new drugs to treat NAFLD, many mechanism studies and drug trials have been conducted, but no specific drug has yet emerged (Dai et al., 2023). In this study, we found that PD alleviated high-fat diet-induced obesity and non-alcoholic fatty liver disease by inhibiting intestinal fat absorption. This study also provides new insights into subsequent fat recycling along the gut-liver axis.

## 2 Materials and methods

### 2.1 Animal experiments

This study used C57/BL mice (19.2 ± 1.4 g) and leptin-deficient mice (OB) obtained from the Experimental Animal Center of Jinan University (Guangzhou, China). The animal living environment was maintained under controlled temperature (22°C ± 1°C), humidity (50%), and light (12 h light/12 h dark). All animal experiments were approved by the Animal Ethics Committee of Jinan University. A high-fat diet (HFD) was fed, consisting of 20% carbohydrates, 35% protein, and 45% fat (60% of total calories according to D12492; Research Diets Inc.). The PD drugs were obtained from the MCE Company (HY-N1411), and the feeding standard was 10 mg/kg/day.

### 2.2 Serological testing and fecal lipid testing

The mice were kept in a fasting and water-free state for 16 h. We then collected the fresh orbital blood, centrifuged the blood at 6,000 ×g for 20 min, and placed it in a −80°C refrigerator for long-term storage. The mouse total triglyceride (TG) (F001-1-1) and total cholesterol (TC) (F002-1-1) were purchased from the Nanjing Jiancheng Bioengineering Institute and tested according to the instructions. The insulin enzyme-linked immunosorbent assay (ELISA) detection kit was purchased from the Abcam Company (ab277390), and the IL-1βelisa detection kit was purchased from the Beyotime Company (PI301). The IL-6 (ab222503) ELISA detection kits were purchased from Abcam. All operations were performed according to the instructions. The insulin resistance model (HOMA-IR) was performed with reference to previous studies (Brown et al., 2019). The method of collecting feces was to collect feces from the colorectum of the mice at the end of the experiment. The free fatty acids (FFA) (A042-2-1) were purchased from the Nanjing Jiancheng Bioengineering Institute and tested according to the instructions.

### 2.3 Glucose tolerance test and insulin resistance test

After the mice were fasted for 16 h, a glucose tolerance test (GTT) was performed, and a 2 g glucose/kg of body weight solution was injected into each mouse. The blood glucose levels were then measured immediately at the 0, 15, 30, 60, and 120 min time points. After 1 week, the mice were fasted for 6 h for insulin resistance testing. A bolus of 0.55 U insulin/kg of body weight was injected into each mouse, and the blood glucose levels were immediately detected at the 0, 15, 30, 60, and 120 min time points. The blood glucose testing used equipment and blood glucose test strips were provided by Sinocare.

### 2.4 Real-time fluorescence quantitative PCR

The fresh mouse organs or tissues were cut into pieces and then ground into powders using liquid nitrogen. The total tissue RNA was then extracted using TRIzol reagent (Ambion). A reverse transcription kit (VAZYME Company, R211-01) was further used to prepare a cDNA template. Amplification products were labeled using SYBR (YEASEN, 10222ES60). RT-PCR was performed using the PCR system of Bio-rad. The relative mRNA levels were calculated using the comparative threshold cycle method. The primer sequences are shown in Table 1.

**TABLE 1:** Primer information table.

Gene name	Forward	Reverse
Cd36	TTC​CAG​CCA​ATG​CCT​TTG​C	TGG​AGA​TTA​CTT​TTT​CAG​TGC​AG
Mttp	ATA​CAA​GCT​CAC​GTA​CTC​CAC​T	TCT​CTG​TTG​ACC​CGC​ATT​TTC
Npc1L1	CTC​TGC​CCT​CTG​CAA​TGC​TC	GAA​CAG​GCT​GCC​GAG​TCT​T
ApoB	GCT​CAA​CTC​AGG​TTA​CCG​TGA	AGG​GTG​TAC​TGG​CAA​GTT​TGG
Abca1	GCT​TGT​TGG​CCT​CAG​TTA​AGG	GTA​GCT​CAG​GCG​TAC​AGA​GAT
Actb	GGC​TGT​ATT​CCC​CTC​CAT​CG	CCA​GTT​GGT​AAC​AAT​GCC​ATG​T

### 2.5 Tissue staining

The fresh livers, intestines, and adipose tissues were fixed using 4% paraformaldehyde for 24 h and then embedded using OCT, followed by being sliced into 8-um thick slices using a Thermo Fisher Scientific microtome and stored at −20°C for later use.

H&E staining: The cut tissue sections were then washed three times using phosphate buffer solution (PBS), placed in the hematoxylin solution for 1 min for staining, washed with PBS three more times, placed in the eosin solution for 1 min, washed with PBS three times again, and then xylene was used to make them clear. The sections were then sealed using neutral gum. Photos were then taken with a Leica microscope. Oil Red O staining: The cut tissue sections were washed three times with PBS, placed in the Oil Red O solution for staining for 1 min, washed again with PBS three times, placed in the hematoxylin solution for 1 min, washed again with PBS three times, sealed with glycerol gelatin, and a Leica microscope was used to take the photos. ImageJ software was used to obtain the statistics on the oil red area.

### 2.6 Western blot analysis

A radioimmunoprecipitation assay buffer (RIPA lysis buffer) was used to extract the intestinal epithelial tissue proteins, and a bicinchoninic (BCA) protein assay kit was used to determine the protein concentration (Beyotime, Shanghai, China). The membrane was then blocked using 5% BSA for 2 h. The membrane was then incubated with p-AMPKα (Thr172) antibody (cell signal technology, 2535S, United States) and AMPK (cell signal technology, 2532S, United States) (1:1000) at 4°C for 24 h. The beta-actin antibody (Gene Tex Corporation, GTX109639, United States) (1:5000) level was then assessed as a loading control, and the secondary antibody Anti-Rabbit-lgG (H + L) antibody (Jackson Corporation, 711-035-152, United States) (1: 5000) was bound to the primary antibody for 2 h at room temperature. The signals were then captured using a chemiluminescence imaging analysis system (Sinsitech, China, Minichemi 610). The intensity of each band was quantitatively analyzed using ImageJ software.

### 2.7 Cell culture

The cryopreserved colon cancer cell line (Caco2 cells) was revived and passaged for 2 passages. It was then transferred to a six-well plate with approximately 1,000 cells per well and then removed after 24 h of growth. PD was added to the well plate to a final concentration of 4 μM, and then the dissolving reagent PBS solution was added to the control group. The cells were then cultured again for 12 h and removed for the Oil Red O staining. The specific operation was as follows. The cells were washed three times with PBS, then 1 mL of the Oil Red O solution was added for staining for 1 min. The cells were washed three times again with PBS, and pictures were obtained using a Leica microscope. ImageJ software was to obtain the statistics of the Oil Red O area. The Caco2 cell culture conditions were 10% fetal bovine serum + Dulbecco’s Modified Eagle Medium (DMEM) high glucose (25.5 mM) medium. The ambient temperature was set to 37°C, and the carbon dioxide concentration was 5%. All cell operations were completed in a sterile clean bench.

### 2.8 Statistical analysis

Data are expressed as the mean ± standard error of the mean (SEM). Furthermore, significant differences were determined by performing a t-test with least significant difference (LSD) *post hoc* tests, and statistical significance was set at *p* < 0.05.

## 3 Results

### 3.1 Platycodon D improved glucose metabolism in mice fed a high-fat diet

**FIGURE 1:**
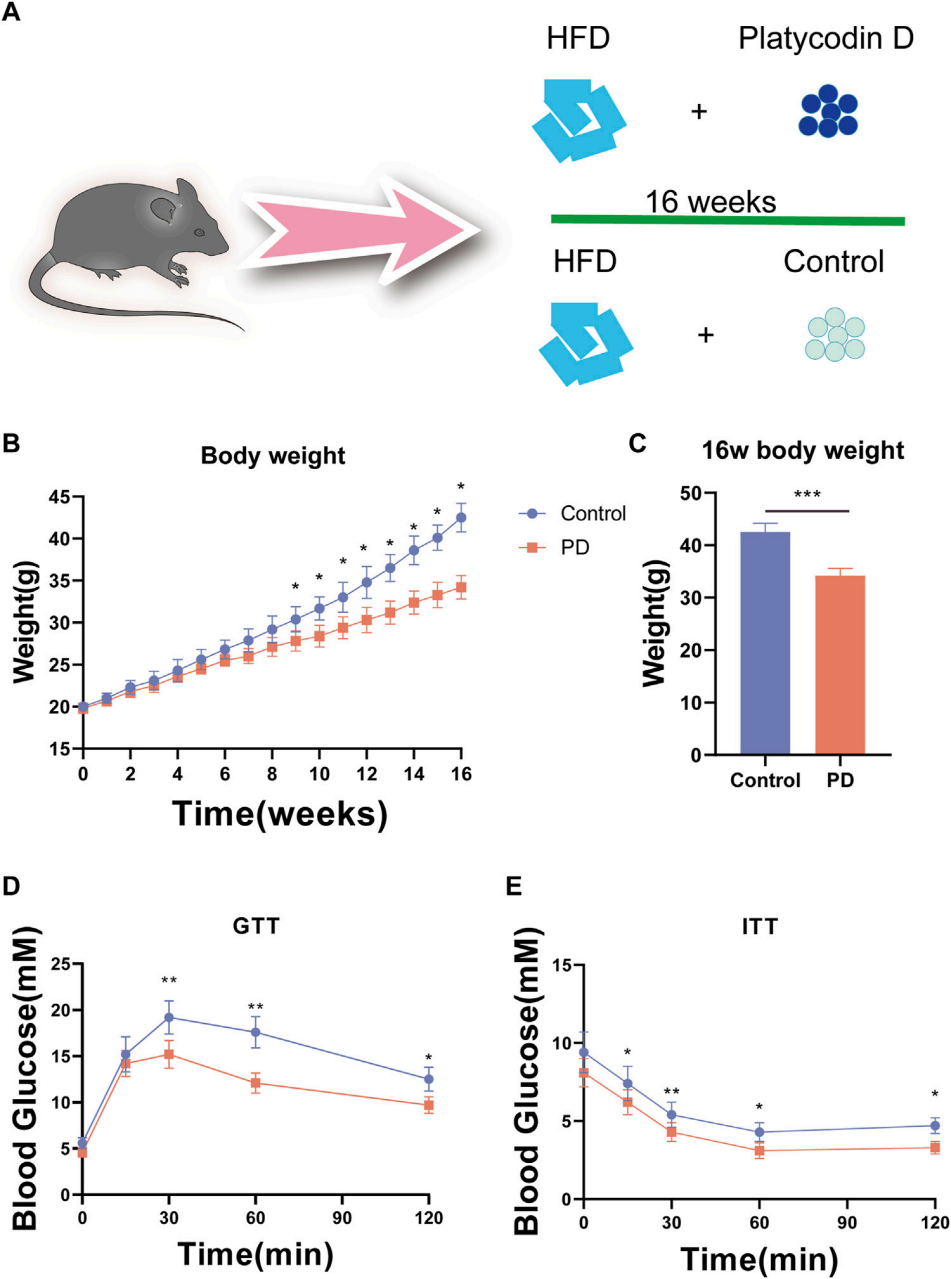
**(A)** Experimental model diagram of the high-fat diet mice. **(B)** Mouse body weights. **(C)** Body weight statistics of mice fed a high-fat diet for 16 weeks. **(D)** GTT in mice. **(E)** ITT in mice. n = 5, *p < 0.05, **p < 0.01,***p < 0.001.

To verify the effect of platycodin D on glucose metabolism in mice. We used platycodin D to feed mice on a high-fat diet and conducted a 16-week experimental observation. The control group used the solvent control (Figure 1A). The results showed that the body weights of the high-fat diet mice in the platycodin D group were significantly lower than that of the control group at week 10. This phenotype was maintained until the end of the experiment. At 16 weeks, the high-fat diet mice in the platycodin D group had significantly lower body weights than those of the control group. Compared with the control group, the body weights were significantly reduced by approximately 20% (*p* < 0.05) (Figure 1B, C). Changes in body weight may change the body’s glucose and lipid metabolism phenotype. Therefore, we performed a glucose tolerance test (GTT) and an insulin tolerance test (ITT). The results showed that compared with the control group, platycodin D significantly improved the glucose tolerance of mice on a high-fat diet, especially 30 min after the glucose injection when the blood glucose levels of the mice in the platycodin D group were significantly lower than that of the control group (*p* < 0.05) (Figure 1D). In terms of insulin tolerance, the insulin sensitivities of the mice in the platycodin D group were significantly higher than that of the control group (*p* < 0.05) (Figure 1E). These results indicated that platycodin D improved glucose metabolism in mice fed a high-fat diet, and this may have been related to weight loss.

### 3.2 Platycodin D improved lipid metabolism in mice fed a high-fat diet

**FIGURE 2:**
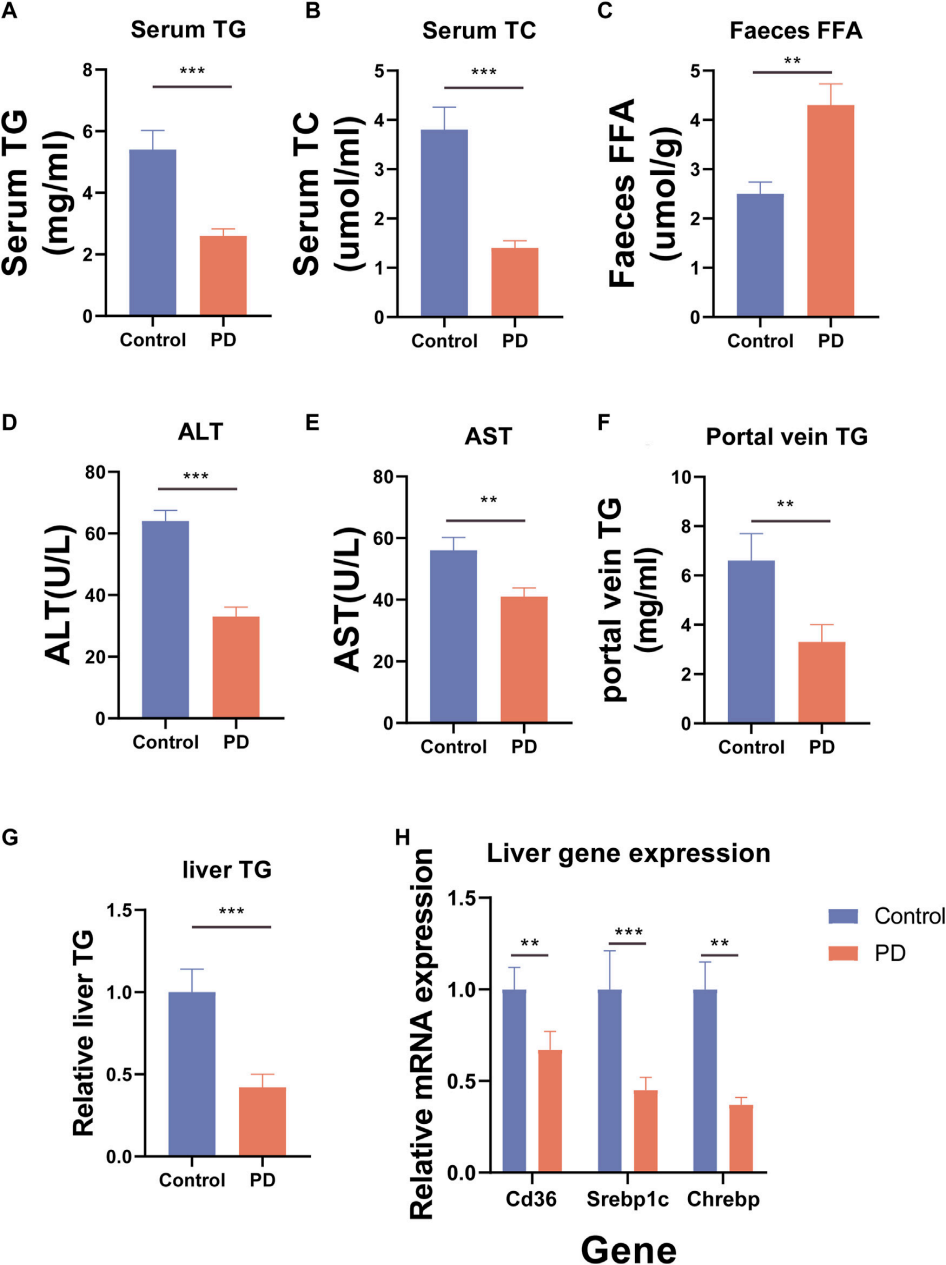
**(A)** Mouse serum TG. **(B)** Mouse serum TC. **(C)** Mouse fecal FFA. **(D)** Mouse serum ALT. **(E)** Mouse serum AST. **(F)** Mouse portal vein TG. **(G)** Mouse liver TG. **(H)** Expression of lipid absorption genes in the mouse livers. n = 3–4, *p < 0.05,**p < 0.01,***p < 0.001.

To further observe the effect of platycodin D on lipid metabolism in mice, we further examined the serum lipid (total triglyceride (TG) and total cholesterol (TC)) levels of the mice as well as the free fatty acids (FFA) in the feces. The results showed that compared with the control group, the high-fat diet mice fed platycodin D had significantly lower serum TG and TC levels and also excreted more FFA in their feces (*p* < 0.05) (Figures 2A–C). These results suggested that platycodin D may modulate intestinal lipid excretion in response to a high-fat diet, thereby causing the serum lipid levels in mice to lower. To further verify our conjecture; we also tested the portal vein TG contents and liver damage markers (alanine transaminase (ALT) and aspartate aminotransferase (AST)) of the mice and found that platycodin D reduced the portal vein TG levels and treated liver damage caused by high-fat diet feeding (*p* < 0.05) (Figure 2D–F). In other words, platycodin D improved the body’s metabolic phenotype by reducing intestinal lipid absorption. Since lipid accumulation in the body first occurs in the liver, we tested the TG contents of the livers, the liver lipid absorption genes (CD36, Srebp1c, and Chrebp), and the TG levels. The results showed that compared with the control group, platycodin D reduced high-fat liver TG in mice fed a high-fat diet, and this change may have been mediated by reducing the expression levels of the liver lipid absorption genes (CD36, Srebp1c, and Chrebp) (*p* < 0.05) (Figure 2G, H). These results indicated that platycodin D improved lipid metabolism in the mice fed a high-fat diet.

### 3.3 Platycodin D reduced lipid absorption in the intestine and liver

**FIGURE 3:**
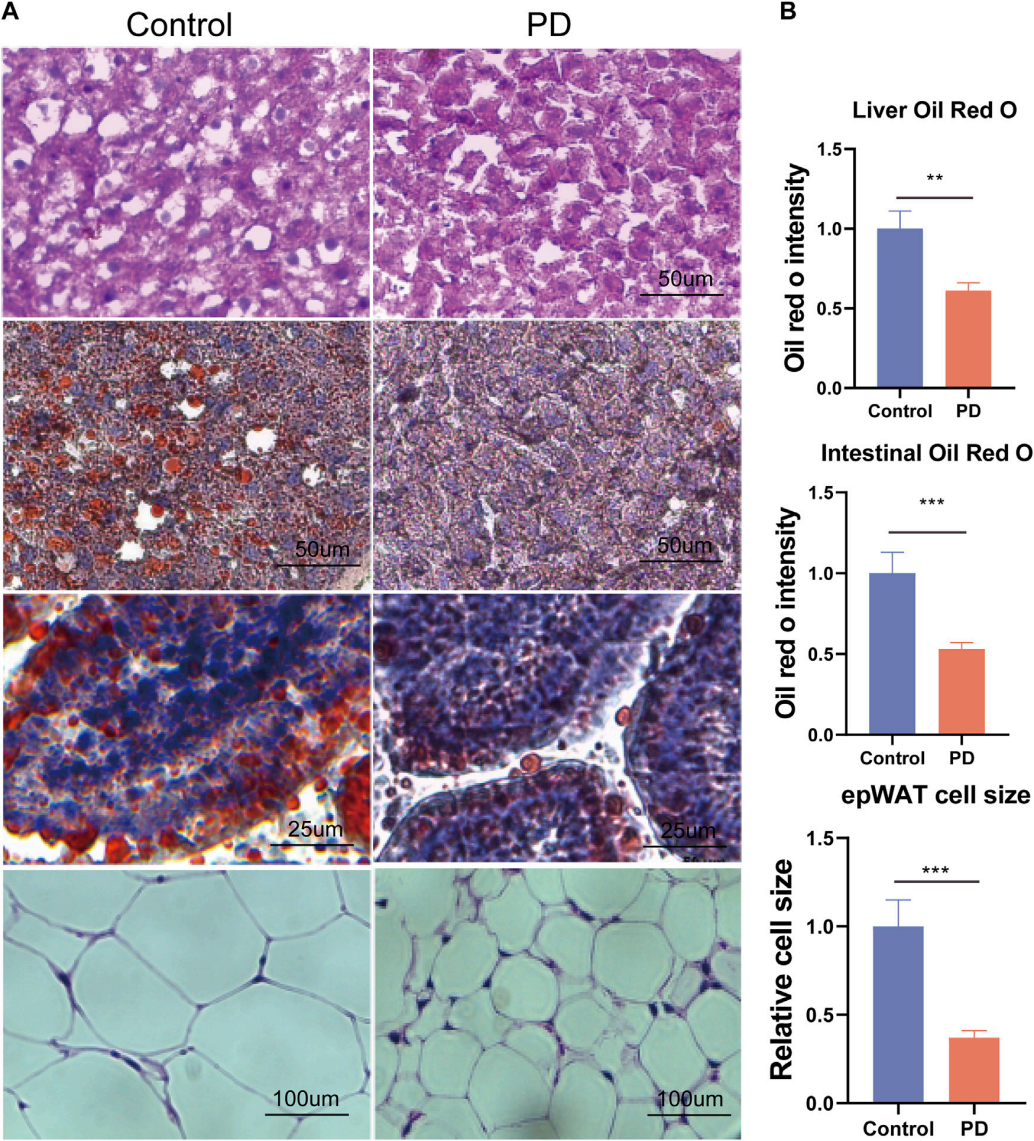
**(A)** H&E staining of the mouse livers, Oil Red O staining of the livers, Oil Red O staining of the intestines, and H&E staining of fat, and **(B)** the related statistical diagram. n = 3–4, *p < 0.05,**p < 0.01,***p < 0.001.

**FIGURE 4:**
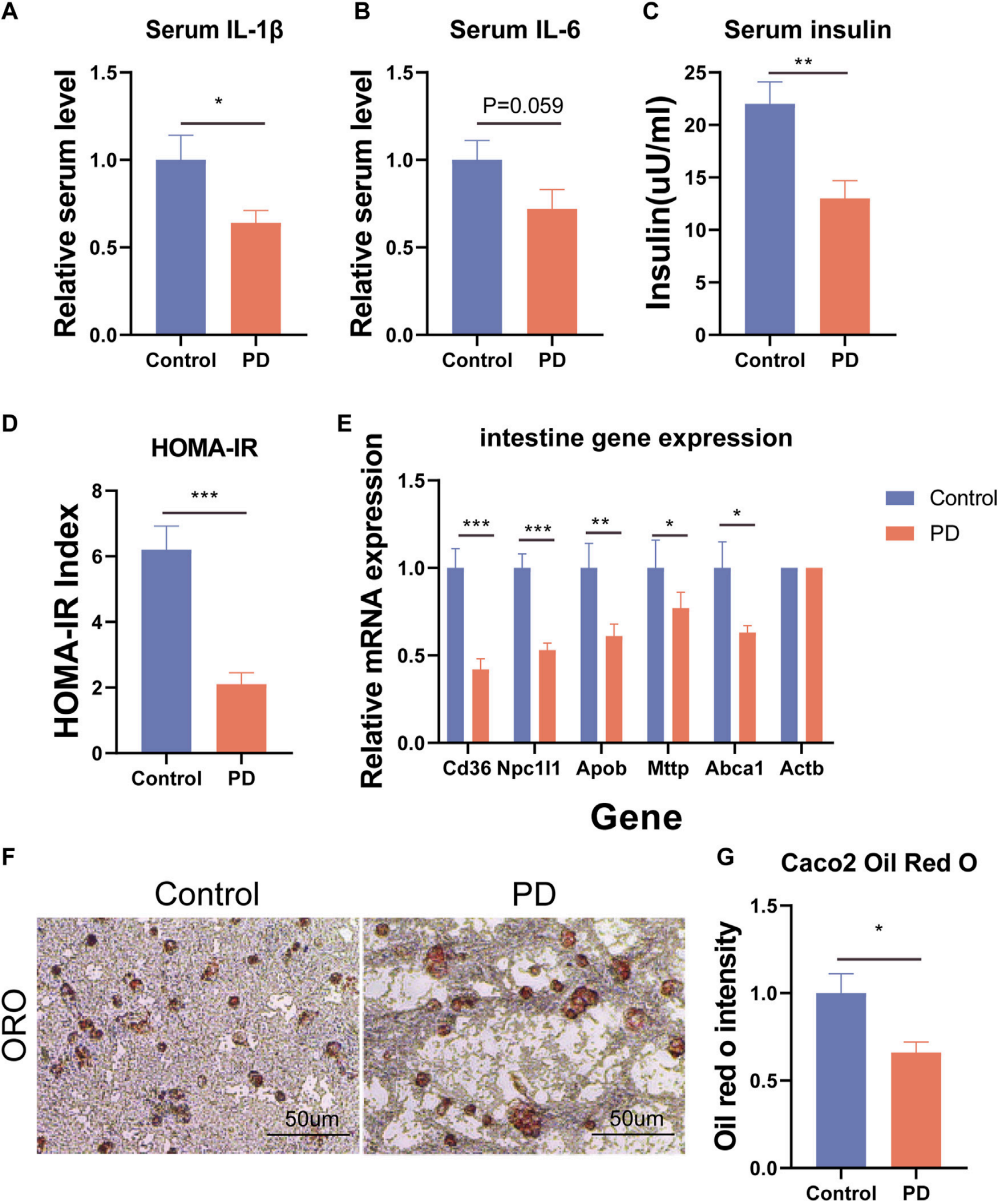
**(A)** Mouse serum IL-1β. **(B)** Mouse serum IL-6. **(C)** Mouse serum insulin levels. **(D)** Insulin resistance model HOMA-IR. **(E)** Expression levels of intestinal lipid absorption genes. **(F)** Oil Red O staining of the Caco2 cells, and **(G)** the lipid proportion statistics. n = 3, *p < 0.05,**p < 0.01,***p < 0.001.

To further explore how platycodin D reduced the lipid levels in mice, we speculated that platycodin D may reduce intestinal and liver lipid absorption, thereby causing a decrease in blood lipid levels in mice. To verify our conjecture, we performed H&E staining and Oil Red O staining on the livers, intestines, and visceral fat (epididymal fat (epWAT)) of the mice. The results showed that the livers of mice in the control group showed obvious vacuolar degeneration, hepatocyte swelling, and liver fat accumulation. In addition, the Oil Red O staining and fat H&E staining results of the jejunum of the control group showed that the intestinal lipid absorption of the mice in the control group increased and the epididymal adipocytes increased significantly. However, mice fed a high-fat diet in the platycodin D group significantly reversed this phenomenon. Specifically, platycodin D reduced the intestinal lipid absorption, thereby improving liver lipid accumulation and vacuolar degeneration, while fat accumulation in epididymal fat was reduced (*p* < 0.05) (Figure 3A, B). These results indicated that platycodin D reduced lipid accumulation in the liver and adipose tissues by reducing intestinal lipid absorption, thereby reducing the body weights and metabolic phenotypes. To explore the changes in inflammation levels and insulin resistance caused by platycodin D by reducing intestinal lipid absorption, we detected the expression of inflammatory factors and insulin in the serum of mice fed a high-fat diet. The results showed that compared with the control group, the serum inflammatory factors (IL-1β and IL-6) and insulin levels of mice in the platycodin D group were significantly reduced (*p* < 0.05) (Figure 4A–C). In addition, the insulin resistance model (HOMA-IR) was significantly lower (*p* < 0.05) (Figure 4D). This demonstrated that the platycodon D group had improved serum inflammation and insulin resistance in mice. To further verify that the phenotypic improvement was caused by reducing intestinal lipid absorption, we detected the expression levels of lipid absorption genes in the mouse intestinal epithelial cells. The results showed that the platycodin D group had reduced expressions of the lipid absorption genes (CD36, NPC1L1, Apob, Mttp, and Abca1) (*p* < 0.05) (Figure 4E). The above results indicated that platycodin D reduced lipid absorption in the intestines and livers, thereby reducing inflammation and lipid accumulation in mice.

### 3.4 Platycodin D may have reduced intestinal lipid uptake by activating the AMPK pathway

**FIGURE 5:**
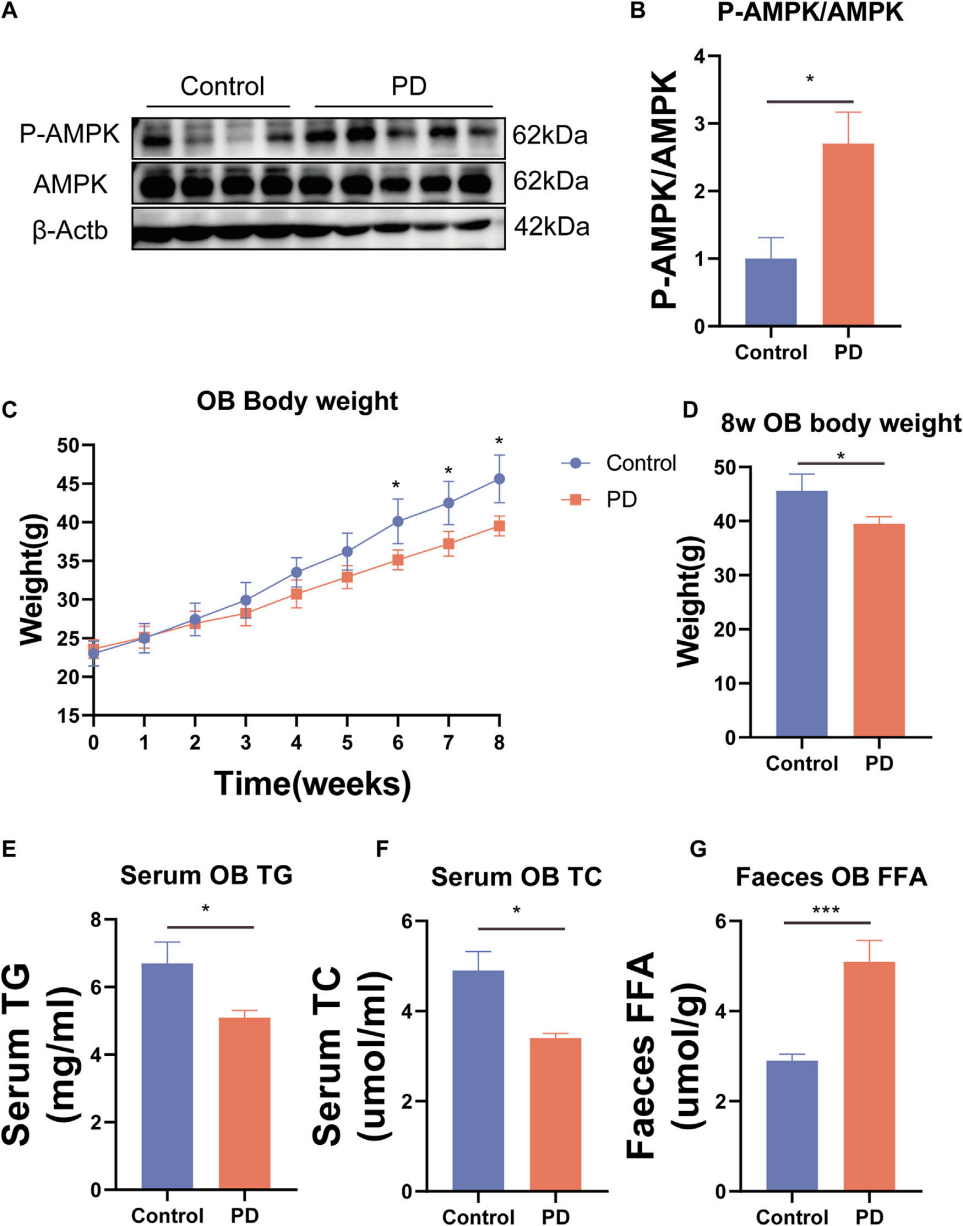
**(A)** Expression of the AMPK phosphorylated protein and **(B)** statistical graph. **(C)** Weight gain of the OB mice. **(D)** Body weight comparison of the OB mice at 8 weeks. **(E)** Serum TG in the OB mice. **(F)** Serum TC in the OB mice. **(G)** Fecal FFA of the OB mice. n = 3, *p < 0.05,**p < 0.01,***p < 0.001.

To further confirm that platycodin D could reduce intestinal lipid absorption, we added platycodin D into the Caco2 cells for co-culture and then performed Oil Red O staining. The results showed that platycodin D reduced lipid accumulation in the Caco2 cells (*p* < 0.05) (Figure 4F, G). Previous studies have reported that intestinal lipid absorption is related to AMPK phosphorylation levels. Therefore, we detected the intestinal AMPK phosphorylation levels of the mice. The results showed that compared with the control group, platycodin D significantly increased the intestinal AMPK phosphorylation levels (*p* < 0.05) (Figure 5A, B). This indicated that platycodin D may reduce lipid absorption by activating intestinal AMPK phosphorylation.

### 3.5 Platycodin D improved lipid metabolism in the leptin-deficient mice

**FIGURE 6:**
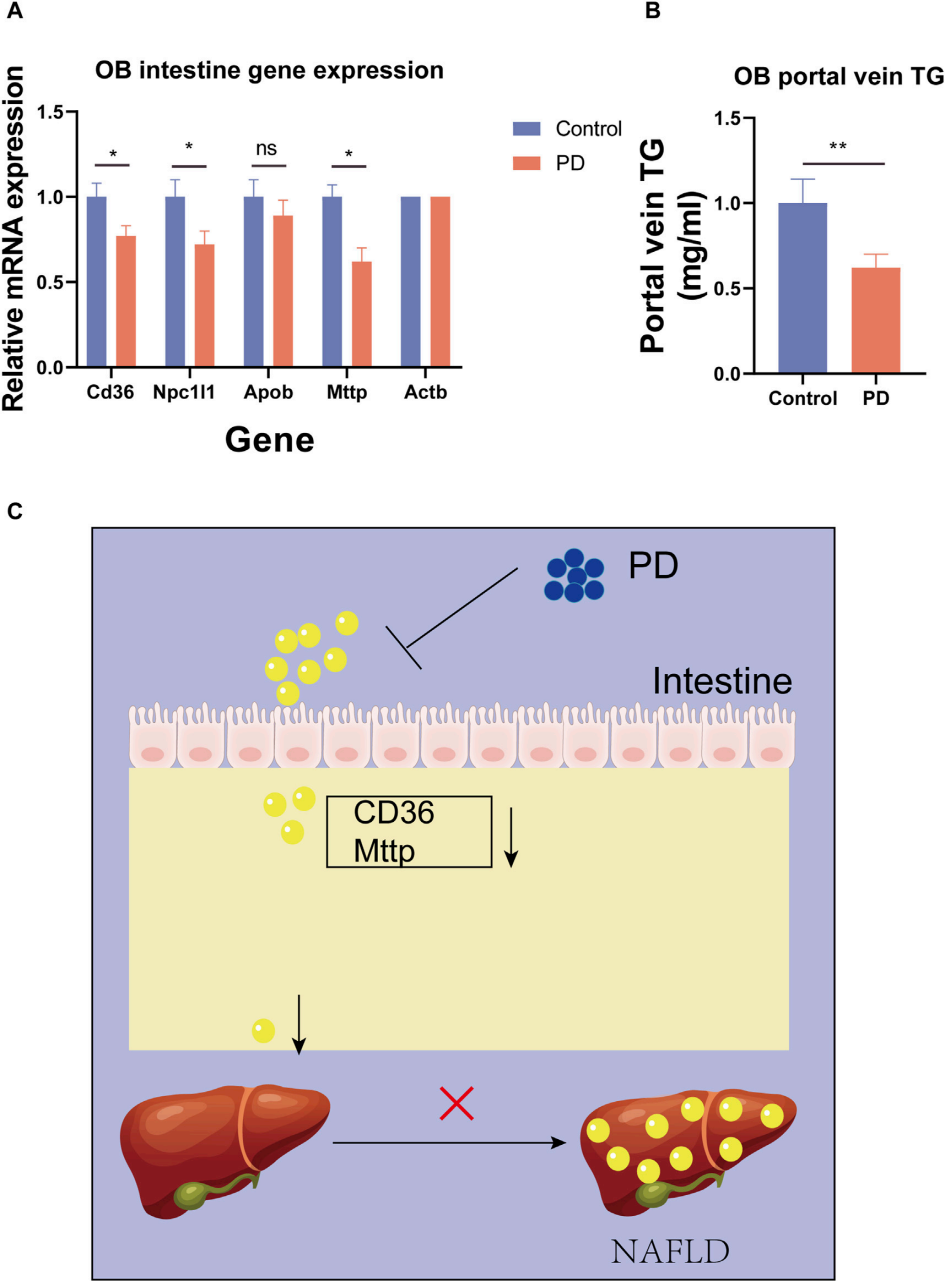
**(A)** Expression levels of the intestinal lipid absorption genes in the OB mice. **(B)** Portal TG levels in the OB mice. **(C)** Schematic diagram of the effects of PD on the mouse intestines. n = 3, *p < 0.05,**p < 0.01,***p < 0.001.

To further verify the ability of platycodin D to regulate lipids in mice, we used platycodin D to feed leptin-deficient mice (OB). The results showed that compared with the control group, platycodin D significantly reduced body weights after feeding the OB mice (*p* < 0.05) (Figure 5C, D). Consistent with the high-fat diet mice, platycodin D reduced serum lipid (TG and TC) levels while increasing the fecal lipid (FFA) excretion levels (*p* < 0.05) (Figure 5E–G). We further examined the portal vein glycerol levels and intestinal lipid absorption gene expression levels in the OB mice. The results showed that compared with the control group, platycodin D reduced the absorption of TG in the portal veins of the OB mice (*p* < 0.05) (Figure 6B). In addition, platycodin D also reduced the expression of intestinal lipid absorption genes (CD36, NPC1L1, and Mttp) in the OB mice (*p* < 0.05) (Figure 6A). These results further confirmed that platycodin D reduced lipid accumulation in the body by reducing intestinal lipid absorption (Figure 6C).

## 4 Discussion

As a natural plant extract, PD is widely used in the treatment of various diseases, including intestinal diseases, vascular diseases, diabetes, obesity, endocrine diseases, and inflammation control (Arai et al., 1997; Han et al., 2002; Xu et al., 2005; Chen et al., 2016; Guo et al., 2021; Wang et al., 2022). It has been reported that PD can alleviate lipid accumulation in fatty acid-treated HepG2 cells (Xu et al., 2024b). We hypothesized that this ability to reduce lipid accumulation results from the reduced uptake of external fatty acids. Therefore, we have reason to believe that for the body, reducing intestinal fatty acid absorption can reduce the occurrence and development of various metabolic syndromes.

In our study, we found that PD improved glucose metabolism, including body weight, glucose tolerance, and insulin resistance, in mice fed a high-fat diet. Our findings are consistent with previous research (Han et al., 2002). In addition, this beneficial effect, we believe, was due to weight loss. Therefore, we further found that PD improved lipid metabolism in mice fed a high-fat diet. Specifically, PD reduced blood lipid levels (TG, TC, and FFA) in mice fed a high-fat diet. In addition, we found that the expressions of lipid absorption genes (CD36) in the intestine and liver of the PD-treated high-fat diet mice were significantly reduced. Additionally, it was found that the portal vein TG levels of high-fat diet mice treated with PD were significantly reduced, which indicated that PD may reduce the impact of high-fat diet by regulating intestinal lipid absorption. Furthermore, we observed the lipid accumulation in the livers, intestines, and epididymal fat of the high-fat mice and found that PD reduced the lipid accumulation in the livers, intestines, and epididymal fat of the high-fat mice. We hypothesized that PD may reduce blood lipid levels by reducing intestinal lipid absorption, thereby affecting lipid accumulation in the liver and adipose tissue. Therefore, we used a colon cancer cell line (Caco2) to test our hypothesis. Surprisingly, PD reduced lipid accumulation in Caco2 cells, which verified our hypothesis that PD inhibits intestinal lipid absorption. Many studies have shown that the intestinal lipid absorption capacity may be related to AMPK protein activation (Li et al., 2016; Ran et al., 2021). We subsequently found that the intestinal AMPK phosphorylation levels of high-fat diet mice treated with PD increased, which suggested that PD may reduce intestinal lipid absorption by activating intestinal AMPK phosphorylation levels, thereby reducing liver lipid accumulations. Subsequently, to verify our findings in the high-fat diet mice, we used leptin-deficient mice (OB) for further studies. The results were consistent with those in the mice fed a high-fat diet. PD reduced blood lipid levels and portal vein TG levels in OB mice. Additionally, PD still reduced the expression level of the intestinal lipid absorption gene (CD36).

The intestine plays a very important and irreplaceable role in the body. As an important part of intestinal function, intestinal absorption provides nutritional support and an energy source for the body. Previous researchers have focused on the effect of PD on colon inflammation (Guo et al., 2021), while ignoring the regulatory effect of PD on the small intestine. This study found that PD had an anti-metabolic syndrome effect on the regulation of lipid absorption in the small intestine. Fat digestion and absorption play a crucial role in maintaining energy homeostasis and supporting basic physiological functions (Omer and Chiodi, 2024). Proper use of the role of PD in the intestine can benefit people with metabolic diseases. Currently, the primary treatment for many overweight people and people with metabolic syndrome is bariatric surgery, and this surgery has long-lasting complications and side effects on the body. As an alternative, we can provide PD to reduce intestinal lipids. We have provided new insights into the absorption and thus reduction of lipid accumulation in the liver and adipose tissue. This will expand the application scope of PD disease treatment.

## 5 Conclusion

In summary, in this study, we found that PD improved glucose and lipid metabolism in high-fat diet mice, which involved PD activating intestinal AMPK phosphorylation and thereby reducing lipid absorption, which will reduce lipid accumulation in the liver and adipose tissue. Systemic lipid accumulation was also reduced accordingly, thereby improving metabolic syndrome.

## Data Availability

The raw data supporting the conclusions of this article will be made available by the authors, without undue reservation.
